# Supplementary material for: Ethical review of COVID-19 research in the Netherlands; a mixed-method evaluation among medical research ethics committees and investigators
Source: PLoS One. 2021 Jul 23;16(7):e0255040. doi: 10.1371/journal.pone.0255040 (PMC8301608; doi:10.1371/journal.pone.0255040)
Supplement: S1 File — (PDF) [file pone.0255040.s001.pdf]

Questionnaire emergency procedure version 1.5 dated 18-5-2020
(principal) investigators

Consent form

National evaluation of the emergency procedure ethics committees/CCMO for the assessment of SARS-CoV-2 research proposals

- I have read the information letter. I have also been able to ask questions. My questions have been adequately answered. I had enough time to decide whether to participate.
- I know that participation is voluntary. I also know that I can decide at any time not to participate or to stop participating in the study. I do not have to give a reason for doing so.
- I consent to the collection and use of my data in the manner and for the purposes set out in the information letter.
- I give permission for my data to be stored at METc VUmc for 5 years after this research.

I wish to participate in this study.

□ Yes
□ No

I consent to being approached for an interview for this study in the manner and for the purposes set out in the information letter.

□ Yes
□ No

Name: (please fill in if we may approach you for participation in a (group) interview)

E-mail address: (please fill in if we may approach you for participation in a (group) interview)

Questionnaire emergency procedure version 1.5 dated 18-5-2020
(principal) investigators

Questionnaire

Please fill in the questionnaire about the research on SARS-CoV-2, **which is subject to the WMO.**

General questions:

1. What is your position?
   - Principal investigator
   - Executive researcher
   - Other, namely

2. How many research proposals relating to SARS-CoV-2 that are subject to the WMO have you submitted to the ethics committee/CCMO?
   - 1
   - 2
   - 3 or more

3. Have you submitted any other research to the ethics committee/CCMO in the past five years in addition to the recently submitted research involving SARS-CoV-2 that is subject to the WMO?
   - No
   - Yes, once
   - Yes, two to five times
   - Yes, more than five times
   - N/A

Submission process:

4. Did you consult with the ethics committee about your submission prior to submitting your study on SARS-CoV-1?
   - Yes
   – No

5. Does the ethics committee to which you submitted your SARS-CoV-2 related study have an emergency procedure for the assessment of SARS-CoV-2 related research?
   – Yes
   – No
   – I do not know

6. Before submitting your study, were you aware of any emergency procedure for the assessment of SARS-CoV-2 related research?
   - Yes
   – No

7. Was the emergency procedure clear to you before you submitted your research proposal?
   - Yes
   - No
   - N/A

   Please explain:

8. Which documents were required for the assessment of your research on SARS-CoV-2 that is subject to the WMO?
   - The same documents as required for regular research that is subject to the WMO
   - Fewer documents than required for regular research that is subject to the WMO
   - More documents than required for regular research that is subject to the WMO
   - I do not know

   Please explain:

<u>The assessment:</u>

9. Did you feel that the ethics committee was more lenient with regard to the requirements that are normally set for the documents submitted before the research file is declared complete?
   - Much more lenient
   - More lenient
   - No difference
   - Less lenient
   - Much less lenient
   - I don’t know, I have not submitted research that is subject to the WMO before

Please explain:

10. Did you feel that the ethics committee took the urgency of the research into account?
    - Yes
    - No

Please explain:

11. Were the comments of the ethics committee clear?
    - Very clear
    - Clear
    - Unclear
    - Very unclear

12. Do you think the ethics committee made enough distinction between substantive comments and administrative comments?
    - Yes
    - No

Please explain:

13. Did you notice any difference in the ethics committee's assessment of your SARS-CoV-2 study compared to earlier research that was subject to the WMO?
    - Yes
    - No
    - N/A, I have not submitted research subject to the WMO before

Please explain:

14. For each of the following points, please indicate whether you feel that these aspects have been weighed differently in the assessment of your SARS-CoV-2 protocol(s) compared to regular protocols?

	Yes, different/ No not different/ Don't know, not submitted before	Explanation
Burden of test subjects in relation to scientific value		
Legal aspects		
Privacy aspects		
Methodological aspects		
Ethical principles		
Administrative issues		
Information for test subjects		
Otherwise, namely		

<u>Review period:</u>

15. What is your perception of the overall assessment period of the ethics committee of your SARS-CoV-2 study which is subject to the WMO?
    - Very short
    - Short
    - Not long, not short

- Long
- Very long

Please explain:

16. Do you feel that the assessment of your research on SARS-CoV-2, which is subject to the WMO, was faster than the assessment of regular research which is subject to the WMO?
    - Yes
    - No
    - I do not know

Please explain:

Satisfaction:

17. In general, how satisfied are you with the evaluation of your research proposal on SARS-CoV-2 that is subject to WMO?
    - Very satisfied
    - Satisfied
    - Dissatisfied
    - Very dissatisfied

18. Which aspects of the assessment of your research on SARS-CoV-2 that is subject to the WMO are you satisfied with?

19. Which aspects of the assessment of your research on SARS-CoV-2 that is subject to the WMO are you less satisfied with?

20. If you have submitted research that is subject to the WMO to the METC/CCMO more than once, are you more or less satisfied with the assessment of your research involving SARS-CoV-2 compared to the earlier assessment(s)?
    - Much more satisfied
    - More satisfied
    - Equally satisfied
    - Less satisfied
    - Much less satisfied
    - N/A, I have not submitted research that is subject to the WMO before

21. In your opinion, which points went better than in previous regular assessments of research that is subject to the WMO?

22. In your opinion, which points went less well than in previous regular assessments of research that is subject to the WMO?

23. Which points of improvement can you mention in connection with the assessment of your research on SARS-CoV-2 which is subject to the WMO??

Lastly

24. Do you have any other remarks and/or suggestions?
