# Supplementary material for: Ethical review of COVID-19 research in the Netherlands; a mixed-method evaluation among medical research ethics committees and investigators
Source: PLoS One. 2021 Jul 23;16(7):e0255040. doi: 10.1371/journal.pone.0255040 (PMC8301608; doi:10.1371/journal.pone.0255040)
Supplement: S2 File — (PDF) [file pone.0255040.s002.pdf]

Questionnaire emergency procedure version 1.7 dated 11-5-2020
Ethics committee (METC/CCMO)

Consent form

National evaluation of the emergency procedure ethics committees/CCMO for the assessment of SARS-CoV-2 research proposals

- I have read the information letter. I have also been able to ask questions. My questions have been adequately answered. I had enough time to decide whether to participate.
- I know that participation is voluntary. I also know that I can decide at any time not to participate or to stop participating in the study. I do not have to give a reason for doing so.
- I consent to the collection and use of my data in the manner and for the purposes set out in the information letter.
- I give permission for my data to be stored at METc VUmc for 5 years after this research.

I wish to participate in this study.

□ Yes
□ No

I consent to being approached for an interview for this study in the manner and for the purposes set out in the information letter.

□ Yes
□ No

Name: (please fill in if we may approach you for participation in a (group) interview)

E-mail address: (please fill in if we may approach you for participation in a (group) interview)

Questionnaire emergency procedure version 1.7 dated 11-5-2020
Ethics committee (METC/CCMO)

Questionnaire

Depending on your answer, certain questions will be skipped.

If you cannot answer a question, please choose the option 'I don't know' or consult a colleague.

General questions:

1. What is the name of your ethics committee?

    - 1
    - 2
    - 3
    - 4
    - 5
    - 6
    - 7
    - 8
    - 9
    - 10
    - 11
    - 12
    - 13
    - 14
    - 15
    - 16
    - 17
    - 18
    - Other, namely

2. What is your position
    - Chair person
    - Member of ethics committee, namely (position within ethics committee)
    - Secretary
    - Other, namely

Working method:

3. Has your ethics committee set up an emergency procedure with regard to SARS-CoV-2 research?
    - Yes, through to question 9
    - No, through to question 4

4. What is the reason that your ethics committee does not have an emergency procedure? (several answers are possible)

- No emergency protocols expected
- No advantage expected compared to regular procedure
- Time constraints
- Other, namely
- I do not know

Please explain:

5. Have any protocols involving SARS-CoV-2 been discussed in the regular procedure?
   - Yes
   - No
   - I do not know

6. How many protocols related to SARS-CoV-2 have been discussed in the regular procedure? (until 1 May 2020)
   - Subject to the WMO: (number/N/A)
   - Not subject to the WMO: (number/N/A)
   - I do not know

7. Have these SARS-CoV-2 protocols discussed in the regular procedure been processed faster than usual?
   - Yes
   - No
   - N/A
   - I do not know

8. Have any protocols been submitted to another ethics committee that did have an emergency procedure?
   - Yes
   - No
   - I do not know

   Continue with question 43

9. Is this emergency procedure intended for research that is subject to the WMO and/or research that is not subject to the WMO?
   - Research that is subject to the WMO
   - Research that is not subject to the WMO
   - Both research subject as well as research not subject to the WMO
   - I do not know

10. Who assesses protocols **that are subject to the WMO** that are discussed in the emergency procedure? (several answers are possible)
    - Full committee

- Emergency committee
- Executive Board
- Chair and secretary
- Otherwise, i.e.
- N/A
- I do not know

11. Who assesses protocols **that are not subject to the WMO** that are discussed in the emergency procedure? (several answers are possible)
    - Full committee
    - Emergency committee
    - Executive Board
    - Chair and secretary
    - Otherwise, i.e.
    - N/A
    - I do not know

12. If there is an Emergency Sub-Committee, which members make up the Emergency Sub-Committee? (more than one answer is possible)
    - Chair person
    - Vice chair person
    - Ethicist
    - Methodologist
    - Lawyer
    - Paediatrician
    - WMO physician
    - Pharmacist
    - Data protection officer
    - Other members, namely:
    - Depending on the protocol, but at least the following members:
    - N/A
    - I do not know

13. Does the non-WMO assessment also include a substantive test or is there only an assessment of whether the study is subject to the WMO or not?
    - Assessment of the WMO requirement only
    - Substantive test
    - N/A
    - I do not know

14. What documents are required for the assessment of research that is **subject to the WMO** in the emergency procedure?
    - The same documents as those required for regular research that is subject to the WMO
    - Fewer documents than required for regular research that is subject to the WMO

- More documents than are required for regular research that is subject to the WMO
- N/A
- I do not know

Please explain:

15. Which documents are required for the assessment of research that is **not subject to the WMO** in the emergency procedure?
    - The same documents as those required for regular research that is not subject to the WMO
    - Fewer documents than required for regular research that is not subject to the WMO
    - More documents than are required for regular research that is not subject to the WMO
    - N/A
    - I do not know

    Please explain:

16. To what extent is your ethics committee lenient with regard to declaring the research file complete/under consideration when it does not yet fully meet the requirements set in the regular procedure?
    - Very lenient
    - Lenient
    - Not lenient
    - Not al all lenient
    - I do not know

    Please explain:

17. Did your ethics committee have any external support/consultation during the drafting of the emergency procedure? (more than one answer is possible)
    - Yes, from/with the CCMO
    - Yes, from/with another ethics committee
    - Yes, otherwise:
    - No
    - I do not know

18. In what way has your ethics committee shared information about the emergency procedure with investigators? (more than one answer is possible)
    - Website ethics committee
    - E-mail to investigators
    - No information shared
    - Other, namely:
    - I do not know

Assessment deadlines:

Questionnaire emergency procedure version 1.7 dated 11-5-2020
Ethics committee (METC/CCMO)

19. On average, within what time frame did the **first assessment** of research that is **subject to the WMO** take place after a research file was submitted?
    - Within 1 day
    - Within 1-2 days
    - Within 2-3 days
    - Within 3-7 days
    - Within 1-2 weeks
    - After 2 weeks
    - I do not know

20. When did your ethics committee assess the first emergency protocol on SARS-CoV-2?
    - Research subject to the WMO: date xx-xx-xxxx / N/A
    - Research not subject to the WMO: date xx-xx-xxxx / N/A
    - I do not know

21. How many research proposals had been assessed in the emergency procedure by 1 May?
    - Subject to the WMO: (number/N/A)
    - Not subject to the WMO: (number/N/A)
    - I do not know

22. Have any protocols involving SARS-CoV-2 been discussed in the regular procedure?
    - Yes
    - No
    - I do not know

23. How many protocols involving SARS-CoV-2 have been discussed in the regular procedure? (until 1 May 2020)
    - Subject to the WMO: (number/N/A)
    - Not subject to the WMO: (number/N/A)
    - I do not know

24. Have these SARS-CoV-2 protocols discussed in the regular procedure been processed faster than usual?
    - Yes
    - No
    - N/A
    - I do not know

25. How much faster do you estimate that the assessment of your ethics committee in the emergency procedure of research that is subject to the WMO is compared to the regular assessment?
    - Not faster
    - 0-1 week faster
    - 1-2 weeks faster

- 2-4 weeks faster
- > 4 weeks faster
- I do not know

26. How long was the <u>total</u> assessment time of emergency protocols subject to the WMO from submission to approval in days? (up to 1 May 2020)
    - Average: (number of days)
    - Minimum (shortest assessment period): (number of days)
    - Maximum (longest assessment period): (number of days)
    - I do not know

Experiences:

27. To what extent was it difficult to find committee members willing to assess research files in the emergency procedure?
    - Not difficult at all
    - Not difficult
    - Difficult
    - Very difficult
    - I do not know

    Please explain:

28. In general, what is your opinion about the quality of the submitted research files in the SARS-CoV-2 emergency procedure compared to regular submissions?
    - Much worse
    - Worse
    - No difference
    - Better
    - Much better
    - I do not know

    Please explain:

It is possible that your ethics committee has been more or less strict in the assessment of the protocols in the emergency procedure due to the urgency of the research. Please explain this in the following questions.

29. How strict did your ethics committee generally judge the emergency protocols for SARS-CoV2 compared to regular protocols?
    - Much less strict
    - Less strict
    - As strict as regular

- Stricter
- Much stricter
- I do not know

Please explain:

30. For each of the following points, please indicate whether you feel that these aspects have been weighed differently in the assessment of your SARS-CoV-2 protocol(s) compared to regular protocols?

	Yes, different/ No not different/	Explanation
Burden of test subjects in relation to scientific value		
Legal aspects		
Privacy aspects		
Methodological aspects		
Ethical principles		
Administrative issues		
Information for test subjects		
Otherwise, namely		

31. How much weight does your committee attach to the urgency of the SARS-CoV-2 research in the assessment of the research proposal?
    - No weight al all
    - Not much weight
    - Some weight
    - Much weight
    - I do not know

32. Do you consider the quality of the assessment of your ethics committee of research on SARS-CoV-2 in the emergency procedure different from that of regular research?
    - Much better
    - Better
    - No difference
    - Not as good
    - Much less good
    - I do not know

33. Do you expect that the assessment of the remaining protocols will be delayed due to the prioritisation of SARS-CoV-2 related protocols?
    - Yes

- No
- I do not know

Please explain:

34. Have other tasks of your ethics committee (e.g. education/consultation) been dropped or postponed because of the emergency procedure for the review of SARS-CoV-2 related protocols?
    - Yes
    - No
    - I do not know

    Please explain:

35. Did your ethics committee need (more) external support/consultation <u>in setting up</u> the emergency procedure? (more than one answer is possible)
    - Yes, from/with the CCMO
    - Yes, from/with other ethical committees
    - Yes, other, namely
    - No
    - I do not know

    Please explain:

36. Did your committee need (more) substantive external support/consultation during <u>the assessment</u> of the SARS-CoV-2 research files?
    - Yes
    - No
    - I do not know

    Please explain:

37. What do you consider to be the strengths of the emergency procedure of your ethics committee compared to the regular procedure?

38. What do you consider to be the weaknesses of the emergency procedure of your ethics committee compared to the regular procedure?

39. What has your ethics committee learnt from the emergency procedure?

40. In general, how satisfied are you with the emergency procedure of your ethics committee for the assessment of SARS-CoV-2 research?
    - Very satisfied
    - Satisfied

- Neutral
- Unsatisfied
- Very unsatisfied

Expectations for the future:

41. For each of the following points, please indicate whether you expect the regular assessment of your ethics committee to change as a result of experiences with the emergency procedure for SARS-CoV-2 research?

	Yes, different/ No not different/	Explanation
Burden of test subjects in relation to scientific value		
Legal aspects		
Privacy aspects		
Methodological aspects		
Ethical principles		
Administrative issues		
Information for test subjects		
Otherwise, namely		

42. Please indicate for each of the following points whether you expect the procedures of your ethics committee to change as a result of experiences with the emergency procedure for SARS-CoV-2 research?

	Yes/no	Explanation
Method of meeting (physical or online)		
Frequency of meetings		
Composition of committee		
Assessment deadlines		
Activities secretariat		
Otherwise, namely		

Lastly

43. Do you have any other remarks and/or suggestions?
