# Supplementary material for: Ethical review of COVID-19 research in the Netherlands; a mixed-method evaluation among medical research ethics committees and investigators
Source: PLoS One. 2021 Jul 23;16(7):e0255040. doi: 10.1371/journal.pone.0255040 (PMC8301608; doi:10.1371/journal.pone.0255040)
Supplement: S3 File — (PDF) [file pone.0255040.s003.pdf]

Vragenlijst spoedprocedure versie 1.5 d.d. 18-5-2020
(hoofd)onderzoekers

Toestemmingsformulier

Landelijke evaluatie spoedprocedure METCs/CCMO voor de beoordeling van SARS-CoV-2 onderzoeksvoorstellen

- Ik heb de informatiebrief gelezen. Ook kon ik vragen stellen. Mijn vragen zijn voldoende beantwoord. Ik had genoeg tijd om te beslissen of ik meedoe.
- Ik weet dat meedoen vrijwillig is. Ook weet ik dat ik op ieder moment kan beslissen om toch niet mee te doen of te stoppen met het onderzoek. Daarvoor hoef ik geen reden te geven.
- Ik geef toestemming voor het verzamelen en gebruiken van mijn gegevens op de manier en voor de doelen die in de informatiebrief staan.
- Ik geef toestemming om mijn gegevens nog 5 jaar na dit onderzoek te bewaren bij de METc VUmc.

Ik wil meedoen aan dit onderzoek.

□ ja
□ nee

Ik geef toestemming om mij te benaderen voor een interview voor dit onderzoek op de manier en voor de doelen die in de informatiebrief staan.

□ ja
□ nee

Naam: (graag invullen als we u mogen benaderen voor deelname aan een (groeps)interview)

E-mailadres: (graag invullen als we u mogen benaderen voor deelname aan een (groeps)interview)

Vragenlijst spoedprocedure versie 1.5 d.d. 18-5-2020
(hoofd)onderzoekers

Vragenlijst

Graag invullen over het door u ingediende **WMO-plichtige** onderzoek m.b.t. SARS-CoV-2.

Algemene vragen:

1. Wat is uw functie?
   - Hoofdonderzoeker
   - Uitvoerend onderzoeker
   - Anders, namelijk

2. Hoeveel **WMO-plichtige** onderzoeksvoorstellen m.b.t. SARS-CoV-2 heeft u ingediend bij de METC/CCMO?

   - 1
   - 2
   - 3 of meer

3. Heeft u de afgelopen vijf jaar naast het recent ingediende WMO-plichtige onderzoek m.b.t. SARS-CoV-2 nog een **ander WMO-plichtig** onderzoek ingediend bij de METC/CCMO?
   - Nee
   - Ja, één keer
   - Ja, twee tot vijf keer
   - Ja, meer dan vijf keer
   - nvt

Indieningsproces:

4. Heeft u voorafgaand aan het indienen van uw onderzoek m.b.t. SARS-CoV-2 overlegd met de toetsingscommissie over uw indiening?
   - Ja
   – Nee

5. Heeft de toetsingscommissie waar u uw onderzoek m.b.t. SARS-CoV-2 heeft ingediend een spoedprocedure voor de beoordeling van SARS-CoV-2 gerelateerd onderzoek?
   – Ja
   – Nee
   – Weet ik niet

6. Was u voordat u uw onderzoek indiende op de hoogte van een eventuele spoedprocedure voor de beoordeling van SARS-CoV-2 onderzoek?
   - Ja
   – Nee

7. Was de spoedprocedure u duidelijk voordat u uw onderzoeksvoorstel indiende?
   - Ja
   - Nee
   - Nvt

Graag toelichten:

8. Welke documenten werden voor de beoordeling van uw WMO-plichtig onderzoek m.b.t. SARS-CoV-2 verplicht gesteld?
   - Dezelfde documenten zoals vereist bij regulier WMO-plichtig onderzoek
   - Minder documenten dan vereist bij regulier WMO-plichtig onderzoek
   - Meer documenten dan vereist bij regulier WMO-plichtig onderzoek
   - Weet ik niet

Graag toelichten:

De beoordeling:

9. Had u het idee dat de toetsingscommissie coulanter omging met de vereisten die normaalgesproken gesteld worden aan de ingediende documenten voordat het onderzoeksdossier compleet wordt verklaard?
   - Veel coulanter
   - Coulanter
   - Geen verschil
   - Minder coulant
   - Veel minder coulant
   - Weet ik niet, niet eerder WMO-plichtig onderzoek ingediend

Graag toelichten:

10. Had u het idee dat de toetsingscommissie rekening hield met de urgentie van het onderzoek?
    - Ja
    - Nee

Graag toelichten:

11. Was het commentaar van de toetsingscommissie duidelijk?
    - Zeer duidelijk
    - Duidelijk
    - Onduidelijk
    - Zeer onduidelijk

12. Vindt u dat er door de toetsingscommissie voldoende onderscheid is gemaakt tussen inhoudelijke commentaarpunten en administratieve commentaarpunten?
   - Ja
   - Nee

   Graag toelichten:

13. Merkte u verschil in de beoordeling van de toetsingscommissie van uw SARS-CoV-2 onderzoek t.o.v. eerder ingediend WMO-plichtig onderzoek?
   - Ja
   - Nee
   - Nvt, niet eerder WMO-plichtig onderzoek ingediend

   Graag toelichten:

14. Geef voor elk van de volgende punten aan of u het idee heeft dat deze aspecten anders gewogen zijn in de beoordeling van uw SARS-CoV-2 protocol(len) in vergelijking met reguliere protocollen?

	Ja, anders/ Nee, niet anders/weet ik niet, niet eerder ingediend	Toelichting
Belasting van proefpersonen in relatie tot de wetenschappelijke waarde		
Juridische aspecten		
Privacy aspecten		
Methodologische aspecten		
Ethische principes		
Administratieve punten		
Voorlichting aan proefpersonen		
Anders, namelijk		

Beoordelingstermijn:

15. Wat is uw perceptie van de totale beoordelingstermijn van de toetsingscommissie van uw WMO-plichtige onderzoek m.b.t. SARS-CoV-2?
   - Zeer kort
   - Kort
   - Niet lang, niet kort
   - Lang
   - Zeer lang

Graag toelichten:

16. Heeft u het idee dat de beoordeling van uw WMO-plichtige onderzoek m.b.t. SARS-CoV-2 sneller is verlopen dan de beoordeling van regulier WMO-plichtig onderzoek?
    - Ja
    - Nee
    - Weet ik niet

    Graag toelichten:

Tevredenheid:

17. Hoe tevreden bent u in het algemeen over de beoordeling van uw WMO-plichtige onderzoeksvoorstel m.b.t. SARS-CoV-2?
    - Zeer tevreden
    - tevreden
    - ontevreden
    - Zeer ontevreden

18. Over welke aspecten m.b.t. de beoordeling van uw WMO-plichtige onderzoek m.b.t. SARS-CoV-2 bent u tevreden?

19. Over welke aspecten m.b.t. de beoordeling van uw WMO-plichtige onderzoek m.b.t. SARS-CoV-2 bent u minder tevreden?

20. Indien u vaker een WMO-plichtig onderzoek bij de METC/CCMO heeft ingediend, bent u meer of minder tevreden over de beoordeling van uw onderzoek m.b.t. SARS-CoV-2 in vergelijking met de eerdere beoordeling(en)?
    - Veel meer tevreden
    - Meer tevreden
    - Gelijk
    - Minder tevreden
    - Veel minder tevreden
    - Nvt, niet eerder WMO-plichtig onderzoek ingediend

21. Welke punten gingen in uw ogen beter dan bij eerdere reguliere beoordelingen van WMO-plichtig onderzoek?

22. Welke punten gingen in uw ogen minder goed dan bij eerdere reguliere beoordelingen van WMO-plichtig onderzoek?

23. Welke verbeterpunten kunt u noemen naar aanleiding van de beoordeling van uw WMO-plichtige onderzoek m.b.t. SARS-CoV-2?

Ten slotte

24. Heeft u nog overige opmerkingen en/of suggesties?
