# Supplementary material for: Ethical review of COVID-19 research in the Netherlands; a mixed-method evaluation among medical research ethics committees and investigators
Source: PLoS One. 2021 Jul 23;16(7):e0255040. doi: 10.1371/journal.pone.0255040 (PMC8301608; doi:10.1371/journal.pone.0255040)
Supplement: S4 File — (PDF) [file pone.0255040.s004.pdf]

Vragenlijst spoedprocedure versie 1.7 d.d. 11-5-2020 (definitieve via Survalyzer verstuurde versie)
Toetsingscommissies (METC/CCMO)

Tostemmingsformulier

Landelijke evaluatie spoedprocedure METCs/CCMO voor de beoordeling van SARS-CoV-2 onderzoeksvoorstellen

- **Ik heb de informatiebrief gelezen. Ook kon ik vragen stellen. Mijn vragen zijn voldoende beantwoord. Ik had genoeg tijd om te beslissen of ik meedoe.**
- **Ik weet dat meedoen vrijwillig is. Ook weet ik dat ik op ieder moment kan beslissen om toch niet mee te doen of te stoppen met het onderzoek. Daarvoor hoef ik geen reden te geven.**
- **Ik geef toestemming voor het verzamelen en gebruiken van mijn gegevens op de manier en voor de doelen die in de informatiebrief staan.**
- **Ik geef toestemming om mijn gegevens nog 5 jaar na dit onderzoek te bewaren bij de METc VUmc.**

Ik wil meedoen aan dit onderzoek.

□ ja

□ nee

Ik geef toestemming om mij te benaderen voor een interview voor dit onderzoek op de manier en voor de doelen die in de informatiebrief staan.

□ ja

□ nee

Naam: (graag invullen als we u mogen benaderen voor deelname aan een (groeps)interview)

E-mailadres: (graag invullen als we u mogen benaderen voor deelname aan een (groeps)interview)

Vragenlijst spoedprocedure versie 1.7 d.d. 11-5-2020 (definitieve via Survalyzer verstuurde versie) Toetsingscommissies (METC/CCMO)

Vragenlijst

Afhankelijk van uw antwoord zullen bepaalde vragen overgeslagen worden.

Wanneer u een vraag niet kunt beantwoorden kies dan voor de optie 'weet ik niet' of overleg met een collega.

Algemene vragen:

1. Wat is de naam van uw toetsingscommissie?

    - 1
    - 2
    - 3
    - 4
    - 5
    - 6
    - 7
    - 8
    - 9
    - 10
    - 11
    - 12
    - 13
    - 14
    - 15
    - 16
    - 17
    - 18
    - Anders, namelijk

2. Wat is uw functie?
    - Voorzitter
    - METc-lid, namelijk (functie binnen METc)
    - Secretaris
    - Anders, namelijk

Werkwijze:

3. Heeft uw toetsingscommissie een spoedprocedure m.b.t. SARS-CoV-2 onderzoek ingesteld?
    - Ja, door naar vraag 9
    - Nee, door naar vraag 4

4. Wat is de reden dat uw toetsingscommissie geen spoedprocedure heeft? (meerdere antwoorden zijn mogelijk)

- Geen spoedprotocollen verwacht
- Geen voordeel t.o.v. reguliere procedure verwacht
- Tijdgebrek
- Anders, namelijk
- Weet ik niet

Graag toelichten:

5. Zijn er protocollen die SARS-CoV-2 betroffen in de reguliere procedure besproken?
   - Ja
   - Nee
   - Weet ik niet

6. Hoeveel protocollen m.b.t. SARS-CoV-2 zijn in de reguliere procedure besproken? (tot 1 mei 2020)
   - WMO-plichtig: (aantal/nvt)
   - Niet-WMO-plichtig: (aantal/nvt)
   - Weet ik niet

7. Zijn deze protocollen m.b.t. SARS-CoV-2 die in de reguliere procedure zijn besproken sneller afgehandeld dan normaal?
   - Ja
   - Nee
   - Nvt
   - Weet ik niet

8. Zijn er protocollen na overleg met uw toetsingscommissie bij een andere toetsingscommissie ingediend die wel een spoedprocedure had?
   - Ja
   - Nee
   - Weet ik niet

   Door naar vraag 43

9. Is deze spoedprocedure bedoeld voor WMO-plichtig en/of niet-WMO-plichtig onderzoek?
   - WMO-plichtig onderzoek
   - Niet-WMO-plichtig onderzoek
   - Zowel WMO-plichtig als niet-WMO-plichtig onderzoek
   - Weet ik niet

10. Wie beoordeelt de **WMO**-plichtige protocollen die in de spoedprocedure worden besproken? (meerdere antwoorden zijn mogelijk)
    - Gehele commissie
    - Spoedsubcommissie

- Dagelijks Bestuur
- Voorzitter en secretaris
- Anders, nl
- Nvt
- Weet ik niet

11. Wie beoordeelt de **niet-WMO**-protocollen die in de spoedprocedure worden besproken? (meerdere antwoorden zijn mogelijk)
    - Gehele commissie
    - Spoedsubcommissie
    - Dagelijks Bestuur
    - Voorzitter en secretaris
    - Anders, nl
    - Nvt
    - Weet ik niet

12. Indien sprake is van een spoedsubcommissie: welke leden maken deel uit van de spoedsubcommissie? (meerdere antwoorden zijn mogelijk)
    - Voorzitter
    - Vice voorzitter
    - Ethicus
    - Methodoloog
    - Jurist
    - Kinderarts
    - WMO-arts
    - Apotheker
    - Functionaris gegevensbescherming
    - Overige leden, namelijk:
    - Afhankelijk van het protocol, maar minimaal de volgende leden:
    - Nvt
    - Weet ik niet

13. Wordt er bij de niet-WMO beoordeling ook een inhoudelijke toets gedaan of wordt er enkel beoordeeld of de studie WMO-plichtig is of niet?
    - Alleen beoordeling WMO-plichtigheid
    - Inhoudelijke toets
    - Nvt
    - Weet ik niet

14. Welke documenten worden voor beoordeling van **WMO-plichtig** onderzoek in de spoedprocedure verplicht gesteld?
    - Dezelfde documenten zoals vereist bij regulier WMO-plichtig onderzoek
    - Minder documenten dan vereist bij regulier WMO-plichtig onderzoek
    - Meer documenten dan vereist bij regulier WMO-plichtig onderzoek

- Nvt
- Weet ik niet

Graag toelichten:

15. Welke documenten worden voor beoordeling van **niet-WMO**-plichtig onderzoek in de spoedprocedure verplicht gesteld?
    - Dezelfde documenten zoals vereist bij regulier niet-WMO-plichtig onderzoek
    - Minder documenten dan vereist bij regulier niet-WMO-plichtig onderzoek
    - Meer documenten dan vereist bij regulier niet-WMO-plichtig onderzoek
    - Nvt
    - Weet ik niet

    Graag toelichten:

16. In hoeverre is uw toetsingscommissie coulant m.b.t. het compleet verklaren/ in beoordeling nemen van het onderzoeksdossier wanneer deze nog niet volledig aan de vereisten die in de reguliere procedure worden gesteld voldoet?
    - Zeer coulant
    - Coulant
    - Niet coulant
    - Helemaal niet coulant
    - Weet ik niet

    Graag toelichten:

17. Heeft uw toetsingscommissie externe ondersteuning /overleg gehad bij het opstellen van de spoedprocedure? (meerdere antwoorden zijn mogelijk)
    - Ja van/met de CCMO
    - Ja van/met een andere METC
    - Ja, anders namelijk:
    - Nee
    - Weet ik niet

18. Op welke manier heeft uw toetsingscommissie informatie over de spoedprocedure gedeeld met onderzoekers? (meerdere antwoorden zijn mogelijk)
    - Website METc
    - E-mail aan onderzoekers
    - Geen informatie gedeeld
    - Anders, namelijk
    - Weet ik niet

Beoordelingstermijnen:

19. Binnen welke termijn vond de <u>eerste beoordeling</u> van **WMO-plichtig** onderzoek gemiddeld plaats nadat een onderzoeksdossier compleet was aangeleverd?

- Binnen 1 dag
- Binnen 1-2 dagen
- Binnen 2-3 dagen
- Binnen 3-7 dagen
- Binnen 1-2 weken
- Na 2 weken
- Weet ik niet

20. Wanneer heeft uw toetsingscommissie het eerste spoedprotocol m.b.t. SARS-CoV-2 beoordeeld?
    - WMO-plichtig onderzoek: datum xx-xx-xxxx / nvt
    - Niet-WMO plichtig onderzoek: datum xx-xx-xxxx / nvt
    - Weet ik niet

21. Hoeveel onderzoeksvoorstellen zijn er tot 1 mei beoordeeld in de spoedprocedure?
    - WMO-plichtig: (aantal/nvt)
    - Niet-WMO-plichtig: (aantal/nvt)
    - Weet ik niet

22. Zijn er ook protocollen die SARS-CoV-2 betroffen in de reguliere procedure besproken?
    - Ja
    - Nee
    - Weet ik niet

23. Hoeveel protocollen m.b.t. SARS-CoV-2 zijn in de reguliere procedure besproken? (tot 1 mei 2020)
    - WMO-plichtig: (aantal/nvt)
    - Niet-WMO-plichtig: (aantal/nvt)
    - Weet ik niet

24. Zijn deze protocollen m.b.t. SARS-CoV-2 die in de reguliere procedure zijn besproken sneller afgehandeld dan normaal?
    - Ja
    - Nee
    - Nvt
    - Weet ik niet

25. Hoeveel sneller schat u dat de beoordeling van uw toetsingscommissie in de spoedprocedure van **WMO-plichtig** onderzoek is in vergelijking met de reguliere beoordeling?
    - Niet sneller
    - 0-1 week sneller
    - 1-2 weken sneller
    - 2-4 weken sneller

- >4 weken sneller
- Weet ik niet

26. Hoe lang was de totale beoordelingstermijn van **WMO-plichtige** spoedprotocollen vanaf indiening tot aan goedkeuring in dagen? (tot 1 mei 2020)
    - gemiddeld: (aantal dagen)
    - minimaal (kortste beoordelingstermijn): (aantal dagen)
    - maximaal (langste beoordelingstermijn): (aantal dagen)
    - Weet ik niet

Ervaringen:

27. In hoeverre was het lastig om commissieleden bereid te vinden om onderzoeksdossiers te beoordelen in de spoedprocedure?
    - Helemaal niet lastig
    - Niet lastig
    - Lastig
    - Heel lastig
    - Weet ik niet

    Graag toelichten:

28. Wat vindt u over het algemeen van de kwaliteit van de ingediende onderzoeksdossiers in de spoedprocedure SARS-CoV-2 in vergelijking met reguliere indieningen?
    - Veel slechter
    - Slechter
    - Geen verschil
    - Beter
    - Veel beter
    - Weet ik niet

    Graag toelichten:

29. Mogelijk is uw toetsingscommissie door de urgentie van het onderzoek strikter danwel minder strikt geweest in de beoordeling van de protocollen in de spoedprocedure. Dit kunt u toelichten in de volgende vragen.

    Hoe strikt heeft uw toetsingscommissie de spoedprotocollen m.b.t. SARS-CoV2 over het algemeen beoordeeld vergeleken met reguliere protocollen?

    - Veel minder strikt
    - Minder strikt
    - Net zo strikt als regulier
    - Strikter

- Veel strikter
- Weet ik niet

Graag toelichten:

30. Geef voor elk van de volgende punten aan of deze aspecten naar uw mening anders gewogen zijn in de beoordeling van SARS-CoV-2 protocollen in vergelijking met reguliere protocollen?

	Ja, anders/ Nee, niet anders	Toelichting
Belasting van proefpersonen in relatie tot de wetenschappelijke waarde		
Juridische aspecten		
Privacy aspecten		
Methodologische aspecten		
Ethische principes		
Administratieve punten		
Voorlichting aan proefpersonen		
Anders, namelijk		

31. Hoe zwaar telt voor uw toetsingscommissie de urgentie van het SARS-CoV-2 onderzoek bij de beoordeling van het onderzoeksvoorstel?
- Helemaal niet zwaar
- Niet zwaar
- Zwaar
- Heel zwaar
- Weet ik niet

32. Vindt u de kwaliteit van de beoordeling van uw toetsingscommissie van onderzoek m.b.t. SARS-CoV-2 in de spoedprocedure anders dan bij regulier onderzoek?
- Veel beter
- Beter
- Geen verschil
- Minder goed
- Veel minder goed
- Weet ik niet

33. Verwacht u dat de beoordeling van de overige protocollen vertraging zal oplopen doordat SARS-CoV-2-gerelateerde protocollen voorrang krijgen?
- Ja
- Nee
- Weet ik niet

Graag toelichten:

34. Zijn er andere taken van uw toetsingscommissie (bijvoorbeeld onderwijs /overleg) weggevallen of uitgesteld vanwege de spoedprocedure voor de beoordeling van SARS-CoV-2 –gerelateerde protocollen?
    - Ja
    - Nee
    - Weet ik niet

    Graag toelichten:

35. Had uw toetsingscommissie behoefte aan (meer) externe ondersteuning/overleg bij het opzetten van de spoedprocedure? (meerdere antwoorden zijn mogelijk)
    - Ja, van/met de CCMO
    - Ja, van/met andere METC's
    - Ja, anders, namelijk
    - Nee
    - Weet ik niet

    Graag toelichten:

36. Had uw toetsingscommissie behoefte aan (meer) inhoudelijke externe ondersteuning/overleg tijdens de beoordeling van de onderzoeksdossiers m.b..t SARS-CoV-2?
    - Ja
    - Nee
    - Weet ik niet

    Graag toelichten:

37. Wat vindt u de sterke kanten van de spoedprocedure van uw toetsingscommissie in vergelijking met de reguliere procedure?

38. Wat vindt u de zwakke kanten van de spoedprocedure van uw toetsingscommissie in vergelijking met de reguliere procedure?

39. Wat heeft uw toetsingscommissie geleerd van de ingestelde spoedprocedure?

40. Hoe tevreden bent u in het algemeen over de spoedprocedure van uw toetsingscommissie voor de beoordeling van SARS-CoV-2 onderzoek?
    - Zeer tevreden
    - Tevreden
    - Neutraal
    - Ontevreden

- Zeer ontevreden

Verwachtingen voor de toekomst:

41. Geef voor elk van de volgende punten aan of u verwacht dat de reguliere beoordeling van uw toetsingscommissie gaat veranderen naar aanleiding van ervaringen met de spoedprocedure m.b.t. SARS-CoV-2 onderzoek?

	Ja, anders /Nee, niet anders	Toelichting
Belasting van proefpersonen in relatie tot de wetenschappelijke waarde		
Juridische aspecten		
Privacy aspecten		
Methodologische aspecten		
Ethische principes		
Administratieve punten		
Voorlichting aan proefpersonen		
Anders, namelijk		

42. Geef voor elk van de volgende punten aan of u verwacht dat de procedures van uw toetsingscommissie gaan veranderen naar aanleiding van ervaringen met de spoedprocedure m.b.t. SARS-CoV-2 onderzoek?

	Ja/nee	Toelichting
Manier van vergaderen (fysiek of online)		
Frequentie vergaderingen		
Samenstelling commissie		
Beoordelingstermijnen		
Werkzaamheden secretariaat		
Anders, namelijk		

Ten slotte

43. Heeft u nog overige opmerkingen en/of suggesties?
