# Supplementary material for: Ethical review of COVID-19 research in the Netherlands; a mixed-method evaluation among medical research ethics committees and investigators
Source: PLoS One. 2021 Jul 23;16(7):e0255040. doi: 10.1371/journal.pone.0255040 (PMC8301608; doi:10.1371/journal.pone.0255040)
Supplement: S5 File — (PDF) [file pone.0255040.s005.pdf]

Topic list interview investigators

Dutch

	Topic	Question
**Indienings proces**	Voorafgaand aan indiening	Heeft u vooraf overleg gehad met de toetsingscommissie over uw indiening?
	Bekendheid procedure	Was u bekend met de spoedprocedure en was deze duidelijk voor u?
	Documenten	Welke documenten waren vereist?
	Kwaliteit indieningen	Was was de kwaliteit van uw indiening t.o.v. reguliere indieningen?
	Administratieve aspecten	Werd er anders omgegaan met administratieve aspecten?
**Beoordelingstermijnen**	Snelheid	Wat is uw mening over de beoordelingstermijnen en waren deze anders dan bij regulier onderzoek?
**Ervaringen**	Kwaliteit beoordeling	Was de kwaliteit van de beoordeling anders dan regulier?
	Tevredenheid	Hoe tevreden was u met de beoordeling vergeleken met de beoordeling van regulier onderzoek?
	Verbeterpunten	Hoe kan de procedure worden verbeterd?

English

	Topic	Question
**Implementation process**	Prior to submission	Did you consult the MREC prior to submission about the procedure?
	Publicity procedure	Did you know about a FTRP and if so, was it clear to you?
	Documents	Which documents were required for review?
	Submission quality	How was the quality of your submission and research proposal compared to regular research?
	Administrative aspects	Were administrative aspects handled differently?
**Review timelines**	Speed	What is your opinion about the length of the review timelines and were these different compared to regular review?
**Review experiences**	Review quality	Did you experience differences in (quality of) the review?
	Satisfaction	How satisfied were you with the review compared to regular review?
	Points of improvement	How can the procedure be improved?
