# Supplementary material for: Ethical review of COVID-19 research in the Netherlands; a mixed-method evaluation among medical research ethics committees and investigators
Source: PLoS One. 2021 Jul 23;16(7):e0255040. doi: 10.1371/journal.pone.0255040 (PMC8301608; doi:10.1371/journal.pone.0255040)
Supplement: S6 File — (PDF) [file pone.0255040.s006.pdf]

Topic list interview MRECs

Dutch

	Topic	Question
**Indienings proces**	Werkwijze	Hoe is de spoedprocedure ingesteld en wanneer?
	Commissie	Wie beoordeelde de protocollen?
	Documenten	Welke documenten waren vereist?
	Centrale regie	Werd er samengewerkt met een centrale COVID-19 commissie?
	Overzicht	Was er een overzicht met vergelijkbare studies beschikbaar?
	Administratieve aspecten	Werd er anders omgegaan met administratieve aspecten?
**Beoordelingster mijnen**	Snelheid	Hoe veel sneller werd er beoordeeld?
	Beïnvloedende factoren	Welke factoren maakten dat de beoordeling sneller kon?
**Ervaringen**	Kwaliteit indieningen	Was de kwaliteit van de indieningen anders dan regulier?
	Kwaliteit beoordeling	Was de kwaliteit van de beoordeling anders dan regulier?
	Beoordelingsapecten	Werden beoordelingsaspecten anders gewogen?
	Sterke punten procedure	Wat beviel goed aan de procedure?
	Zwakke punten procedure	Wat beviel minder goed aan de procedure?
	Lessen voor de toekomst	Welke lessen kunnen we leren ter verbetering van toekomstige beoordelingsprocedures?

English

	Topic	Question
**Implementation process**	Method	How was the FTRP implemented and when?
	Committee	Who exactly reviewed the COVID-19 submissions?
	Documents	Which documents were required for review?
	Central direction	Did you work together with a central coordinating committee?
	Overview	Did you have a good overview of comparable studies?
	Administrative aspects	Were administrative aspects handled differently?
**Review timelines**	Speed	How much faster was the review?
	Influencing factors	Which factors expedited the review?
**Review experiences**	Submission quality	Did you experience differences in quality of the submissions?
	Review quality	Did you experience differences in quality of the review?
	Review aspects	Were some aspects weighted differently?
	Strengths	What did you experience as the strengths of the FTRP?
	Weaknesses	What did you experience as weaknesses of the FTRP?
	Lessons learned	Which lessons can be learned to improve future review?
