# Supplementary material for: Ethical review of COVID-19 research in the Netherlands; a mixed-method evaluation among medical research ethics committees and investigators
Source: PLoS One. 2021 Jul 23;16(7):e0255040. doi: 10.1371/journal.pone.0255040 (PMC8301608; doi:10.1371/journal.pone.0255040)
Supplement: S7 File — (PDF) [file pone.0255040.s007.pdf]

COREQ Checklist (Tong 2007)

Medical ethical review of COVID-19 reserach in the Netherlands;
a mixed-method evaluation among Medical Research Ethics Committees and investigators

No	Item	Guide questions/description	Reported (page no)
	**Domain 1: Research team an reflexivity**		
	Personal characteristics		
1	Interviewer/facilitator	Which author/s conducted the interview or focus group?	page 7 table 1
2	Credentials	What were the researcher's credentials? *E.g. PhD, MD*	page 7 table 1
3	Occupation	What was their occupation at the time of the study?	page 7 table 1
4	Gender	Was the researcher male or female?	page 7 table 1
5	Experience and training	What experience or training did the researcher have?	page 7 table 1
	Relationship with participants		
6	Relationship established	Was a relationship established prior to study commencement?	page 7 table 1, page 22 limitations
7	Participant knowledge of the interviewer	What did the participants know about the researcher? e.*g. personal goals, reasons for doing the research*	page 7 table 1, page 22 limitations
8	Interviewer characteristics	What characteristics were reported about the interviewer/facilitator? e.g. *Bias, assumptions, reasons and interests in the research topic*	page 7 table 1
	**Domain 2: Study design**		
	Theoretical framework		
9	Methodological orientation and Theory	What methodological orientation was stated to underpin the study? *e.g. grounded theory, discourse analysis, ethnography, phenomenology, content analysis*	Page 6, Design
	Participant selection		
10	Sampling	How were participants selected? *e.g. purposive, convenience, consecutive, snowball*	Page 6 data collection

11	Method of approach	How were participants approached? e.*g. face-to-face, telephone, mail, email*	Page 6 data collection
12	Sample size	How many participants were in the study?	Page 9 quantative results and page 12 qualitative results
13	Non-participation	How many people refused to participate or dropped out? Reasons?	Page 7 data collection
	Setting		
14	Setting of data collection	Where was the data collected? e.*g. home, clinic, workplace*	Page 7 data collection
15	Presence of non-participants	Was anyone else present besides the participants and researchers?	Page 8 Data collection
16	Description of sample	What are the important characteristics of the sample? *e.g. demographic data, date*	Table 1
	Data collection		
17	Interview guide	Were questions, prompts, guides provided by the authors? Was it pilot tested?	Table 2a and 2b Topic lists
18	Repeat interviews	Were repeat interviews carried out? If yes, how many?	Page 7 data collection
19	Audio/visual recording	Did the research use audio or visual recording to collect the data?	Page 8 data collection
20	Field notes	Were field notes made during and/or after the interview or focus group?	Page 8 data collection
21	Duration	What was the duration of the interviews or focus group?	Table 1
22	Data saturation	Was data saturation discussed?	Page 22 Discussion
23	Transcripts returned	Were transcripts returned to participants for comment and/or correction?	Page 8 data collection
	**Domain 3: analysis and findings**		
	Data analysis		
24	Number of data coders	How many data coders coded the data?	Page 8 Data analysis
25	Description of the coding tree	Did authors provide a description of the coding tree?	Table 5
26	Derivation of themes	Were themes identified in advance or derived from the data?	Page 8 Data analysis
27	Software	What software, if applicable, was used to manage the data?	Page 8 Data analysis
28	Participant checking	Did participants provide feedback on the findings?	Page 8 data collection
	Reporting		

29	Quotations presented	Were participant quotations presented to illustrate the themes / findings? Was each quotation identified? e.*g. participant number*	Results section
30	Data and findings consistent	Was there consistency between the data presented and the findings?	Results section
31	Clarity of major themes	Were major themes clearly presented in the findings?	Results section
32	Clarity of minor themes	Is there a description of diverse cases or discussion of minor themes?	Results section
