# Supplementary material for: Ethical review of COVID-19 research in the Netherlands; a mixed-method evaluation among medical research ethics committees and investigators
Source: PLoS One. 2021 Jul 23;16(7):e0255040. doi: 10.1371/journal.pone.0255040 (PMC8301608; doi:10.1371/journal.pone.0255040)
Supplement: S8 File — (PDF) [file pone.0255040.s008.pdf]

Dutch quotes

"Wij hebben besloten om een subcommissie van de grote commissie samen te stellen met in ieder geval alle verplichte disciplines… En wij hebben steeds vergaderd als er een of twee protocollen binnen kwamen dan werd er ad hoc een vergadering gepland." (METC vertegenwoordiger 8)

"…wat ook wel scheelde denk ik is dat iedereen thuis zit, dus de externe afspraken buiten de deur waren er voor de mensen niet waardoor ze toch wel snel beschikbaar waren telkens, zeker in het weekend." (METC vertegenwoordiger 1)

"… dat ging eigenlijk heel soepel. Want iedereen zat toch thuis dus dat, ja, ik vond dat ja verbazend soepel gaan." (METC vertegenwoordiger 5)

"Wij namen ook genoegen met een versimpeld dossier bij aanvang. Dus niet het volledige dossier maar protocol, IB, IMPD, dat moest er in ieder geval bij aanvang zijn en de andere documenten konden gefaseerd komen" (METC vertegenwoordiger 4)

"Alle handtekeningen enzo, daar gaan we niet op wachten. Als de essentiële documenten er maar zijn." (METC vertegenwoordiger 1)

"Wij vonden gewoon dat het allemaal compleet moest zijn. Maar laten we zeggen soms had je iets wat minder relevant is voor de inhoudelijke beoordeling dat je zei als het dan maar aan het eind van de week er is. Het moest in ieder geval wel aanwezig zijn voor wij ons definitieve 'ja' vertelden." (METC vertegenwoordiger 6)

"Ik denk dat zij nog steeds ook 90% harder gewerkt en sneller gewerkt hebben dan ze gewend zijn . Alleen de 10% die overblijft die zat nog niet helemaal in die tijdsspanne waarin wij dachten. Wij dachten in uren en dagen maar 10% van wat ze normaal gewend zijn dat zit nog niet in uren en dagen. Dus dat levert nog wel eens wat wrijving op ja." (Onderzoeker 6)

"Ja, dat was bij ons ook het is vooral dat je probeert het hele proces zo efficiënt mogelijk te organiseren en de leden op tijd.. zo'n extra vergadering in stemming… .. te brengen en ervoor te zorgen dat ze de stukken hebben ehm.. en zelf ook als secretaris voorrang geven aan de beoordeling. En daarmee doe je niks af aan de zorgvuldigheid alleen het is … het krijgt gewoon absoluut prioriteit boven andere dingen. Dat is denk ik ook wat de fast-track-procedure beoogde." (METC vertegenwoordiger 9)

"Zodra er covid op stond ging iedereen plotseling in de hoogste versnelling. Ja…en dan denk ik: en ander onderzoek dan? Is dat dan niet belangrijk?" (METC vertegenwoordiger 6)

"De gewone protocollen waren inderdaad iets minder en dat kon je makkelijk inpassen en dan gaf je covid voorrang maar die amendementen dat was bij ons wel en is wel een dingetje." (METC vertegenwoordiger 9)

"Nu mochten we natuurlijk een aantal zaken gewoon digitaal aanleveren en we mochten digitale handtekeningen of een bevestigingse-mail…nou ik denk dat dat heel goed is geweest voor het milieu want het bespaart een enorme lading papieren printen en scannen …en ja, waarom kunnen ze dat niet zo houden?" (Onderzoeker 1)

"Iedereen staat stijf van de stress want he het moet van start" (Onderzoeker 1)

"Ja, als we eerder gestart waren een maand eerder waren we klaar met de studie geweest en nu eh…includeren we 1 patiënt per 2 weken … Nee eh…elke week was grote winst geweest." (Onderzoeker 2)

"Het momentum van de inclusie van deelnemers was natuurlijk ook heel cruciaal want nu gaat het natuurlijk ook alweer heel anders allemaal. Dus we hadden echt een enorme tijdsdruk ja." (Onderzoeker 1)

"Ja elke dag telde wat dat betreft met de piek in patiënten aantallen dus het was voor ons onderzoek ook heel erg urgent." (Onderzoeker 7)

Als je ook kijkt naar de kwaliteit…die is gewoon nog steeds…ik denk dat we nu nog steeds een prima protocol draaien met dezelfde kwaliteit als zeg maar bij een normale procedure dus het lijkt alsof de procedure zonder al te veel verlies van kwaliteit gewoon op deze manier kunt doen (Onderzoeker 2)

"Ik heb dat achteraf bijna als een soort in blessing in disguise ervaren want we hebben in het verleden nog al eens de neiging gehad studies enorm op te tuigen en ingewikkeld te maken en nu waren we toch noodgedwongen om het vrij simpel te houden. En dat is het succes van de studie juist enorm ten goede gekomen. Dat we niet in de verleiding zijn gekomen om het te ingewikkeld te maken. Ja dat heb ik eigenlijk als heel prettig ervaren." (Onderzoeker 7)

"Nou ik denk een stukje efficiëntie in het kort en bondig opschrijven van een protocol daar zit heel veel winst in. Zowel in het schrijven als in het beoordelen. En ik denk dat we allemaal de neiging hebben om er heel veel verhaal omheen te schrijven

om zo compleet mogelijk te zijn. Maar dit dwingt je echt om tot de kern te komen en de essentie te scheiden van al die andere zaken die misschien ja niet nodig zijn." (Onderzoeker 7)

"In het algemeen was er geen verschil in kwaliteit, maar ik had af en toe wel het gevoel van veronderstel dat ze nog 3 weken langer hadden gehad dan was het protocol nog wel wat beter geworden. Sommige dingen waren toch niet helemaal doordacht en dat merk je ook wel nu protocollen weer ingetrokken worden en dat men toch met nieuwe informatie…ja men krijgt steeds meer informatie en dat was denk ik wat ik het moeilijkst vond van het hele covid gebeuren: Er kwam elke dag zoveel nieuwe informatie beschikbaar … Daar heb ik zelf persoonlijk en ook als commissie hebben we daar wel mee geworsteld wat je moest doen met al die nieuwe informatie die over je heen gestort werd." (METC vertegenwoordiger 6)

"En als je je beoordeling niet goed doet … dat komt terug op een negatieve manier. Dus nee, we doen gewoon strak de beoordeling zoals we het anders ook zouden doen. Alleen iets sneller nu." (METC vertegenwoordiger 2)

"Snelheid mag niet ten koste gaan van kwaliteit van de beoordeling. Dat was een belangrijk uitgangspunt." (METC vertegenwoordiger 4)

"Wij hebben inderdaad geen concessies gedaan in de grote lijnen maar uit mijn eigen ervaring met de dingen die ik heb gedaan ben ik toch minder streng dan anders geweest op de inderdaad de dt's en t's, dat je denkt van dit kan mooier dit kan beter." (METC vertegenwoordiger 1)

"Nou, ze hebben ons niet ontzien met het commentaar. Ze hebben gewoon het commentaar gegeven zoals wij dat eigenlijk altijd verwachten min of meer de ene keer is het meer dan de andere. Als ik kijk naar de commentaren dan leidt ik daar uit af dat de beoordeling is gedaan zoals altijd met inachtneming van de basale kwaliteitseisen die we stellen aan elkaar." (Onderzoeker 9)

"Bij de eerste beoordeling kreeg ik 33 vragen en die waren een beetje van dezelfde aard als gewoonlijk. En eh.. het grootste gedeelte daarvan waren wel inhoudelijk goede vragen." (Onderzoeker 3)

"Nou wij begonnen ons op een gegeven punt ook wel zorgen te maken met name over bijvoorbeeld de hoeveelheid bloed die er bij mensen werd afgenomen werd maar op dat moment kwam er eigenlijk een initiatief van het ziekenhuis om een centraal punt te maken waar die onderzoeken allemaal geregistreerd werden en ook gekeken werd wat er uitgevoerd kon worden." (METC vertegenwoordiger 1)

"Ik vond veel overlap en te weinig afstemming. En dat is ook een beetje natuurlijk hoe het landje ook in elkaar steekt. Misschien dat we dat de volgende keer anders zouden moeten doen. Waardoor ook de snelheid van laten we zeggen de epidemie kwam en voordat sommige protocollen waren gestart was ie ook alweer verdwenen. Ja dat is met name met interventiestudies met medicijnen. Die hebben nu allemaal grote problemen om hun N te vullen. Dan hopen ze natuurlijk op een tweede golf, ik hoop het niet daarop trouwens, maar en dat was voorkomen geweest als de handen ineen geslagen waren denk ik." (METC vertegenwoordiger 6)

"Ja, ik denk gewoon een heel duidelijk eh…. Algemeen beleid. Waarin METCs hopelijk ook met elkaar overleggen dat je in zo'n geval bepaalde dingen dus eeeuh uitstelt bijvoorbeeld dus dat je eh een iets minder uitgebreid eerste protocol ofzo beoordeelt en dat je dan later wel kijkt wat er verder nog nodig is." (Onderzoeker 4)

"Iedereen doet z'n eigen dingetje een beetje wat meer centraal aansturen een rol zou kunnen spelen om het nog beter te krijgen. Zeker bij medicatiestudies." (METC vertegenwoordiger 6)

"Het was wel heel duidelijk dat er een spoedprocedure was. En daar stonden ook deadlines op de website… alleen wat ik net al aangaf…dat strookte niet helemaal met wat er in de wandelgangen besproken werd. Wij krijgen de indruk dat… eh…ja de beoordeling sneller zou zijn dan uiteindelijk het geval leek. Dus de deadline voor het indienen stond er heel duidelijk op, alleen hoe snel vervolgens de beóordeling zou zijn, dat stond er niet duidelijk op vermeld. En als je dan alleen alles in de mailbox kunt overhandigen dan blijft het daarna ongrijpbaar wat daarna zeg maar de plan van aanpak gaat zijn met jouw documenten die je hebt ingediend." (Onderzoeker 2)

"Nou, bereikbaarheid van het secretariaat telefonisch was niet heel goed. Dat was…, via e-mail ging het, maar telefonisch was het lastig. Maar goed, als je dan de voorzitter belt dan scheelt dat. Maar die zal dat ongetwijfeld niet altijd leuk vinden als ik hem in de volgende projecten die niet met covid te maken hebben weer op die manier ga bellen." (Onderzoeker 6)

"Wat ik zelf heel storend vond is dat ik iedere keer een ander contactpersoon kreeg. Dus als ik dan een vervolg, een antwoord gaf op een e-mail, dan kreeg ik weer een antwoord van een totaal ander persoon en die ging dan bijvoorbeeld dingen herhalen of opnieuw bevestiging vragen en dat vond ik heel jammer." (Onderzoeker 1)

"Juist die kleine dingetjes allemaal bij elkaar even een telefoontje hier, even een telefoontje daar kijken dat je de juiste mensen op het juiste moment bij elkaar hebt, e-mailtjes opstellen dat kost gewoon heel veel tijd voor het secretariaat." (METC vertegenwoordiger 3)
