# Supplementary material for: Ethical review of COVID-19 research in the Netherlands; a mixed-method evaluation among medical research ethics committees and investigators
Source: PLoS One. 2021 Jul 23;16(7):e0255040. doi: 10.1371/journal.pone.0255040 (PMC8301608; doi:10.1371/journal.pone.0255040)
Supplement: Attachment — Submitted filename: PONE-D-21-05904_reviewer.pdf [file pone.0255040.s009.pdf]

PLOS ONE

Medical ethical review of COVID-19 research in the Netherlands; a mixed-method evaluation among Medical Research Ethics Committees and investigators

--Manuscript Draft--

**Manuscript Number:**	PONE-D-21-05904
**Article Type:**	Research Article
**Full Title:**	Medical ethical review of COVID-19 research in the Netherlands; a mixed-method evaluation among Medical Research Ethics Committees and investigators
**Short Title:**	Medical ethical review of COVID-19 research in the Netherlands
**Corresponding Author:**	Roelie IJkema, MSc<br>Amsterdam UMC - Locatie VUMC: Amsterdam UMC Locatie VUmc<br>Amsterdam, NETHERLANDS
**Keywords:**	ethical review; COVID-19 research
**Abstract:**	Background During the beginning of the COVID-19 pandemic there was urgent need for accelerated review of COVID-19 research by Medical research Ethics Committees (MRECs). In the Netherlands this led to implementation of so-called ‘fast-track-review-procedures’ (FTRPs) to enable a rapid start of urgent and relevant research. The objective of this study was to evaluate FTRPs of MRECs in the Netherlands during the COVID-19 pandemic. Methods and findings An explanatory sequential mixed method study was conducted. Online questionnaires and in-depth interviews were conducted among MREC representatives and investigators of COVID-19 research. In addition, data from a national research registration system was requested. Main outcome measures were differences in timelines, quality of the review and satisfaction between FTRPs and Regular Review Procedures (RRP). The total number of review days was shorter in FTRP (median 10.5) compared to RRPs (median 98.0). Review days attributable to the MRECs also declined in FTRPs (median 8.0 versus 50.0). This shortening can be explained by motivated and flexible ad hoc (sub)committees and secretariats, the sense of urgency of those involved, full priority given to COVID-19 research, regular research put on hold due to COVID-19 measures, the use of online video meetings and administrative leniency. The shorter timelines did not affect the quality of the review. Both MREC representatives as well as investigators were generally satisfied with the review of COVID-19 research. Points of improvement identified were the lack of overview of COVID-19 research, better central collaboration and coordination, and the high workload for MREC members and secretariats. Conclusions To improve future medical ethical review during pandemic situations and beyond we recommend to improve (inter)national collaboration and coordination, to improve communication between MRECs and investigators, to reconsider administrative requirements to the application documents, to make use of digital possibilities and to pay attention to workload of MREC members and secretariats.
**Order of Authors:**	Roelie IJkema, MSc
	MJPA Janssens
	JAM van der Post
	CM Licht
**Additional Information:**	
**Question**	**Response**
**Financial Disclosure**<br><br>Enter a financial disclosure statement that describes the sources of funding for the work included in this submission. Review the submission guidelines for detailed requirements. View published research	The author(s) received no specific funding for this work

articles from *PLOS ONE* for specific examples.<br><br>This statement is required for submission and **will appear in the published article** if the submission is accepted. Please make sure it is accurate.<br><br>**Unfunded studies**<br>Enter: *The author(s) received no specific funding for this work.*<br><br>**Funded studies**<br>Enter a statement with the following details:<br>• Initials of the authors who received each award<br>• Grant numbers awarded to each author<br>• The full name of each funder<br>• URL of each funder website<br>• Did the sponsors or funders play any role in the study design, data collection and analysis, decision to publish, or preparation of the manuscript?<br>• **NO** - Include this sentence at the end of your statement: *The funders had no role in study design, data collection and analysis, decision to publish, or preparation of the manuscript.*<br>• **YES** - Specify the role(s) played.<br><br>* typeset	
**Competing Interests**<br><br>Use the instructions below to enter a competing interest statement for this submission. On behalf of all authors, disclose any competing interests that could be perceived to bias this work—acknowledging all financial support and any other relevant financial or non-financial competing interests.<br><br>This statement is required for submission and **will appear in the published article** if the submission is accepted. Please make sure it is accurate and that any funding sources listed in your Funding Information later in the submission form are also declared in your Financial Disclosure statement.	The authors have declared that no competing interests exist.

Powered by Editorial Manager® and ProduXion Manager® from Aries Systems Corporation

View published research articles from *PLOS ONE* for specific examples.<br><br>**NO authors have competing interests**<br><br>Enter: *The authors have declared that no competing interests exist.*<br><br>**Authors with competing interests**<br><br>Enter competing interest details beginning with this statement:<br><br>*I have read the journal's policy and the authors of this manuscript have the following competing interests: [insert competing interests here]*<br><br>* typeset	
**Ethics Statement**<br><br>Enter an ethics statement for this submission. This statement is required if the study involved:<br><br>• Human participants<br>• Human specimens or tissue<br>• Vertebrate animals or cephalopods<br>• Vertebrate embryos or tissues<br>• Field research<br><br>Write "N/A" if the submission does not require an ethics statement.<br><br>General guidance is provided below. Consult the submission guidelines for detailed instructions. **Make sure that all information entered here is included in the Methods section of the manuscript.**	The Central Committee involving Human Subjects (CCMO) approved the study on 23rd April 2020.<br>All participants were provided with written information about the study and gave informed consent in the online questionnaire.

Powered by Editorial Manager® and ProduXion Manager® from Aries Systems Corporation

**Format for specific study types**<br><br>**Human Subject Research (involving human participants and/or tissue)**<br>• Give the name of the institutional review board or ethics committee that approved the study<br>• Include the approval number and/or a statement indicating approval of this research<br>• Indicate the form of consent obtained (written/oral) or the reason that consent was not obtained (e.g. the data were analyzed anonymously)<br><br>**Animal Research (involving vertebrate animals, embryos or tissues)**<br>• Provide the name of the Institutional Animal Care and Use Committee (IACUC) or other relevant ethics board that reviewed the study protocol, and indicate whether they approved this research or granted a formal waiver of ethical approval<br>• Include an approval number if one was obtained<br>• If the study involved *non-human primates*, add *additional details* about animal welfare and steps taken to ameliorate suffering<br>• If anesthesia, euthanasia, or any kind of animal sacrifice is part of the study, include briefly which substances and/or methods were applied<br><br>**Field Research**<br><br>Include the following details if this study involves the collection of plant, animal, or other materials from a natural setting:<br>• Field permit number<br>• Name of the institution or relevant body that granted permission	
**Data Availability**<br><br>Authors are required to make all data underlying the findings described fully available, without restriction, and from the time of publication. PLOS allows rare exceptions to address legal and ethical concerns. See the PLOS Data Policy and FAQ for detailed information.	Yes - all data are fully available without restriction

A Data Availability Statement describing where the data can be found is required at submission. Your answers to this question constitute the Data Availability Statement and **will be published in the article**, if accepted.<br><br>**Important:** Stating 'data available on request from the author' is not sufficient. If your data are only available upon request, select 'No' for the first question and explain your exceptional situation in the text box.<br><br>Do the authors confirm that all data underlying the findings described in their manuscript are fully available without restriction?	
**Describe where the data may be found in full sentences. If you are copying our sample text, replace any instances of XXX with the appropriate details.**<br><br>• If the data are **held or will be held in a public repository**, include URLs, accession numbers or DOIs. If this information will only be available after acceptance, indicate this by ticking the box below. For example: *All XXX files are available from the XXX database (accession number(s) XXX, XXX.)*.<br>• If the data are all contained **within the manuscript and/or Supporting Information files**, enter the following: *All relevant data are within the manuscript and its Supporting Information files.*<br>• If neither of these applies but you are able to provide **details of access elsewhere**, with or without limitations, please do so. For example:<br><br>*Data cannot be shared publicly because of [XXX]. Data are available from the XXX Institutional Data Access / Ethics Committee (contact via XXX) for researchers who meet the criteria for access to confidential data.*<br><br>*The data underlying the results presented in the study are available from (include the name of the third party*	The data underlying the results presented in the study can be requested from the researchers.

and contact information or URL).<br>• This text is appropriate if the data are owned by a third party and authors do not have permission to share the data.<br><br>* typeset	
Additional data availability information:	

Powered by Editorial Manager® and ProduXion Manager® from Aries Systems Corporation

Manuscript
Click here to access/download;Manuscript;20210219 manuscript total PLOS ONE.docx

Medical ethical review of COVID-19 research in the Netherlands; a mixed-method evaluation among Medical Research Ethics Committees and investigators

Authors:

R IJkema, MJPA Janssens, JAM van der Post, CM Licht

Medical Research Ethics Committee VU medical Center, OZW O8A-07, De Boelelaan 1109, 1081 HV, Amsterdam, the Netherlands

R. IJkema review officer Medical Research Ethics Committee, VU medical center

MJPA Janssens ethicist Medical Research Ethics Committee, VU medical center

JAM van der Post chair Medical Research Ethics Committee, VU medical center

CM Licht manager Medical Research Ethics Committee, VU medical center

Correspondence to: R. IJkema r.ijkema@amsterdamumc.nl

Abstract

Background

During the beginning of the COVID-19 pandemic there was urgent need for accelerated review of COVID-19 research by Medical research Ethics Committees (MRECs). In the Netherlands this led to implementation of so-called 'fast-track-review-procedures' (FTRPs) to enable a rapid start of urgent and relevant research. The objective of this study was to evaluate FTRPs of MRECs in the Netherlands during the COVID-19 pandemic.

Methods and findings

An explanatory sequential mixed method study was conducted. Online questionnaires and in-depth interviews were conducted among MREC representatives and investigators of COVID-19 research. In addition, data from a national research registration system was requested. Main outcome measures were differences in timelines, quality of the review and satisfaction between FTRPs and Regular Review Procedures (RRP). The total number of review days was shorter in FTRP (median 10.5) compared to RRPs (median 98.0). Review days attributable to the MRECs also declined in FTRPs (median 8.0 versus 50.0). This shortening can be explained by motivated and flexible ad hoc (sub)committees and secretariats, the sense of urgency of those involved, full priority given to COVID-19 research, regular research put on hold due to COVID-19 measures, the use of online video meetings and administrative leniency. The shorter timelines did not affect the quality of the review. Both MREC representatives as well as investigators were generally satisfied with the review of COVID-19 research. Points of improvement identified were the lack of overview of COVID-19 research, better central collaboration and coordination, and the high workload for MREC members and secretariats.

Conclusions

To improve future medical ethical review during pandemic situations and beyond we recommend to improve (inter)national collaboration and coordination, to improve communication between MRECs and investigators, to reconsider administrative requirements to the application documents, to make use of digital possibilities and to pay attention to workload of MREC members and secretariats.

Introduction

Since the beginning of 2020, the Netherlands has, like the rest of the world, been confronted with the COVID-19 pandemic caused by the SARS-CoV-2 virus [1]. Mortality, severe morbidity, the unfamiliarity with the virus and the lack of knowledge about adequate treatment, detection, spread and prophylaxis, has led to large-scale and urgent medical scientific research initiatives in order to fill these knowledge gaps. Many of the Medical Research Ethics Committees (MRECs) in the Netherlands (box 1) immediately recognized the need for accelerated review of COVID-19 research, which led to implementation of so-called ‘fast-track-review-procedures’ (FTRPs) to enable a rapid start of urgent and relevant research. However, it is unknown how timelines in these FTRPs exactly differ from those of the regular review procedure (RRP) and if certain review aspects were weighted differently. Also, it is unclear if quality of the review process differs between the FTRP and RRP.

**Box 1 Organisation and regulation of medical ethical review in the Netherlands**
In addition to the central committee of human research (CCMO, supervisor, ethics committee, and competent authority), the Netherlands has 17 accredited Medical Research Ethics Committees (MREC) that review medical scientific research with human subjects [2]. Pursuant to the Dutch law ‘Algemene Wet Bestuursrecht’ (Dutch General Administrative Law Act), a so-called reasonable assessment period of 8 weeks (56 days) normally applies to medical and scientific research subject to the Dutch Wet-medisch-wetenschappelijk-onderzoek-met-mensen (WMO, Medical Research involving Human Subjects Act). The WMO prescribes a specific decision period of 60 days which applies to research with medicinal products [3].

Literature on previous evaluations among MRECs about ethical review of research during epidemics is relatively scarce. The Ethics Committee (EC) of the Henan Provincial People’s Hospital published an

overview of the review times of COVID-19-related research and issues encountered during the assessment [4]. Review processes and timelines of the Médecines Sans Frontieres (MSF) ethics review board (ERB) during the Ebola Virus Disease (EVD) epidemic were described by *Schopper et al.* [5] *Alirol et al* described the review of the WHO ethics review committee (ERC) during the EVD epidemic [6].

The need for ethics committees' preparedness during outbreaks or epidemics was already emphasized earlier. *Saxena et al.* presented six recommendations resulting from a World Health Organization (WHO) workshop with representatives of 29 countries, aimed to identify practical processes and procedures to facilitate ethics review during an infectious disease outbreak [7]. They recommended, for example, that 'a national standard operating procedure (SOP) for emergency response ethical review should be developed and adopted by N(R)ECs and/or in-country competent authority' [7].

The present study evaluates the fast MREC review processes and timelines of COVID-19 related research in the Netherlands, including a description of in-depth insight into experiences of MREC representatives as well as investigators.

Methods

Design

An investigator initiated explanatory sequential mixed method study was conducted. First quantitative data regarding timelines, review quality and satisfaction was collected. As an explanatory follow-up the quantitative results were further ~~explained~~ with in-depth qualitative data [8].

Data collection

In the first quantitative phase, in May 2020, an online questionnaire containing questions about the FTRP, timelines, experiences and satisfaction was sent to chairs and secretariats of all MRECs in the Netherlands. Both chairs and secretaries were invited to fill out the questionnaire. Another questionnaire was sent to all investigators of COVID-19 related research registered with the Competent Authority on 25th of April 2020. In addition, review timelines of these COVID-19 related studies as well as comparable regular studies from 2019 were requested from the national registration system of the Central Committee on Research Involving Human Subjects (CCMO) [9].

In the second qualitative phase, all invitees to the questionnaire were asked to participate in an in-depth group interview. Four semi-structured online group interviews were conducted in June and July 2020 using Microsoft Teams: two group interviews with MREC representatives and two group interviews with COVID-19 investigators. Topic lists with open-ended questions were developed based on questionnaire results. Interviews were led by (anonymous) or (anonymous) in presence of other research team members. Interviews were audio-taped and transcribed verbatim.

Data analysis

Descriptive analyses of the quantitative data were performed using IBM SPSS Statistics 26. An initial code structure based on the topic lists was developed to analyse the qualitative data. After review of the transcripts, the code structure was discussed during research team meetings and

adjusted by modifying (sub)codes. Two researchers (anonymous and anonymous) independently coded all four transcripts using the qualitative research program MAXQDA version 2018.2. Interpretation of codes was discussed until agreement was reached. Decisions made in the discussion sessions were debated during regular research team meetings. All researchers agreed with the final encoded transcripts. Finally, all coded transcripts were analysed with help of the program MAXQDA in order to identify themes [8].

Ethics

This study was approved by the CCMO on 23rd April 2020. All participants were provided with written information about the study and gave informed consent.

Results

Quantitative results

20 chairs and 18 secretariats of all 18 MRECs in the Netherlands were invited to fill out the online questionnaire. 23 fully completed questionnaires were returned, representing 17 of the 18 review committees in the Netherlands (94%). 21 of those 23 MREC respondents (91%), representing 16 of the 17 responding MRECs, implemented a FTRP.

Furthermore, 36 investigators were invited to fill out the questionnaire of which 20 responded (56%). 16 (80%) of those respondents also submitted another research file to an MREC in the RRP in the past 5 years.

Implementation process

7 MRECs established an 'ad-hoc subcommittee' to review the COVID-19 submissions. Other formations that reviewed these submissions were, for example, the full regular committee (10 MRECs) or the daily board (3 MRECs). 19 (90%) of the MREC representatives indicated that it was not difficult (at all) to involve committee members in the FTRP. 3 MRECs indicated they consulted another MREC about their FTRP prior to implementation.

18 (86%) of the MREC representatives indicated that their MREC required submission of the same documents as usual. However, 17 (81%) of the MREC representatives indicated that a certain leniency was applied with regard to the completeness of the initial submission. 9 (45%) of the investigators, however, did not notice any difference in leniency of the required documents compared to the RRP and 2 (10%) indicated even less leniency.

Review timelines

Table 1 shows the median total number of days from complete submission to MREC approval was 10.5 days for COVID-19 research. Thereof, the median number of days attributable to the MRECs was 8.0 days. In comparison, for regular research, the median total number of days from complete submission to MREC approval was 98.0 days with a the median number of days attributable to the

MRECs of 50 days.

16 (80%) investigators indicated that the review of their research went faster compared to regular submissions.

Table 1. Review times for COVID-19 research and regular research

Research files	Total days from complete submission to MREC judgmentMedian (range)	EC review days from complete submission to MREC judgment Median (range)
COVID-19 research (N=28) *[illegible]ment between 13-3-2020 and 20-8-2020 and registered at 25-4-2020* *(2 negative decisions)*	10.5 (1-70)	8.0 (1-56)
Regular research (N=443) *judgment between 13-3-2019 and 20-8-2019 (0 negative decisions)*	98.0 (1-363)	50.0 (1-158)

<u>Review experiences</u>

18 (90%) investigators were (very) satisfied about the review process of their COVID-19 research, as were 19 (90%) of the MREC representatives

17 (85%) investigators felt their MREC took the urgency of the research into account and 16 (76%) of the MREC representatives indicated that the urgency of the research has weighted heavily in their review process. 19 (90%) of the MREC representatives indicated the quality of their review was equal to regular review, but only 13 (62%) indicated their review was ‘as strict as in the regular situation’, 4 (19%) indicated the MREC reviewed ‘less strict’ and 1 (5%) ‘stricter’. Table 2 shows the number of respondents that indicated specific aspects were weighted differently in the FTRP.

There was no consensus among MREC representatives about the quality of the submitted research files in the FTRP: 9 (43%) indicated the quality was ‘equal’, 7 (33%) ‘worse’ , 1 (5%) ‘better’ and the

remainder had no opinion.

5 (24%) of the MREC respondents suggested the review of not COVID-19-related submissions to be delayed due to the priority given to COVID-19 research.

**Table 2. No. of respondents that indicated specific aspects were weighted differently in the FTRP compared to the RRP**		
**Review aspect**	**No. of EC respondents (%)**	**No. of investigators (%)**
Burdening of subjects	0 (0%)	1 (5%)
Legal aspects	2 (10%)	2 (10%)
Privacy aspects	1 (5%)	0 (0%)
Methodological aspects	2 (10%)	4 (20%)
Ethical aspects	1 (5%)	0 (0%)
Subject information	1 (5%)	1 (5%)
Administrative aspects	8 (38%)	6 (30%)

Qualitative results

In total, four semi-structured group interviews were conducted with 10 MRECs representatives and 9 investigators. Interviews ranged in length from circa 45 minutes to 1 hour. Subthemes identified were structured on the basis of the three main themes addressed in the interviews: 1. Implementation process 2. Review timelines and 3. Review experiences.

1. Implementation process

In general, COVID-19 review (sub)committees were composed relatively easy due to a great sense of urgency resulting in great willingness and flexibility of MREC members and secretariats. Working remotely from home and using video conferences probably contributed to a rapid scheduling of review meetings. Other measures taken to speed up the review process were frequent and

accessible contact between MREC representatives and investigators and verbal clarification of the committee's comments. Furthermore, although MRECs largely agreed that the research file had to be complete before approval of a study, a certain leniency concerning the completeness of initial submissions was applied to accelerate the review process. In addition, the obligation to submit a wet signature cover letter for primary submissions was suspended temporarily by the Competent Authority.

2. Review timelines

Although investigators were generally pleased with the review timelines and prioritization of COVID-19 research, they indicated that there was still room for improvement of timelines. Suggestions mentioned were for example giving preliminary feedback shortly after submission and improving cooperation with other stakeholders involved, for example legal departments, medical technology departments and (MRECs of) other participating centres.

MREC representatives also mentioned concerns about delayed appraisal of other research projects by giving full priority to COVID-19 research. In particular, the processing of amendments to regular research was mentioned to be significantly delayed. However, because much regular research was put on hold due to measures taken because of the pandemic, the consequences of this delay were minor.

3. Review experiences

<u>Urgency:</u>

Investigators indicated that fast timelines were crucial to be able to include enough patients, because the patient numbers were expected to decline ~~within a reasonable~~ time. The sense of urgency contributed to more dedication by investigators which also contributed to a faster review process compared to the non-pandemic situation. Investigators regretted, however, that they felt not all stakeholders involved in the research process were equally aware of this urgency or had other

priorities related to the pandemic Delays were particularly noted among legal, medical technology departments and participating centers.

<u>Quality of research file:</u>

Investigators were unanimously satisfied with the quality of their research files which was said to only slightly differ in quality compared to regular research. Some even indicated an improvement compared to regular research because they were forced to keep the protocol simple and focussed due to time pressure. MREC representatives however, mentioned that some research files would probably have improved with a few weeks more preparation time. Amendments to initial research protocols were submitted quickly. This was partly caused by the fact that new knowledge about COVID-19 became available every day and because extra centres had to be included in order to achieve the required inclusion rate.

<u>Quality of review:</u>

Investigators and MREC representatives agreed that the quality of the review was not different from usual. MREC respondents were positive about maintaining quality because carefulness should take precedence over speed, but investigators indicated a clearer distinction between main and side issues would have been desirable.

<u>Collaboration and coordination</u>

Especially among MREC respondents a great need for collaboration and coordination between research projects was experienced. Many MRECs took the feasibility of the research projects into account, but they did not always have good overview of the availability of patients and possibilities in the hospital. MRECs were for example concerned about the considerable amount of human tissues that was collected per patient due to inclusion in multiple studies. On some locations this was solved by a central coordinating committee. But there were also concerns about conducting research that was already carried out elsewhere. It was not easy for MRECs to gain insight into this. Particularly for intervention studies, the short peak of the pandemic made it difficult to include enough participants.

More central management and collaboration between different research groups and MRECs could improve this. Furthermore, the respondents also felt room for improvement with regard to the cooperation and coordination between MRECs. There is a need for a standard procedure that works nationally. Now each MREC implemented its own FTRP.

Communication

In general, there was clear communication about when an application should be submitted and discussed in an MREC meeting. However, according to investigators, it was not clear when a response from the MREC could be expected. Moreover, telephone reachability of the secretariats was poor due to working remotely. This lack of clarity and communication options in an urgent situation led to frustration among investigators. A designated contact person was recommended who can improve communication and limit inconsistency in advice.

Workload:

The FTRP meant a significant change to the working processes at the MREC secretariats. Daily routine changed dramatically overnight and workload increased. Besides the great flexibility that was required of both MREC members as secretaries, there was also social and scientific pressure to process everything very quickly. The ~~little~~ decline in regular work because of temporarily halted research projects helped to make it manageable. Logistics, all remote, were a burden on the secretariats.

Discussion

The objectives of the current research were to identify differences between the FTRP ~~en~~ RRP and to gain further insight into these differences and experiences with the FTRP. Results of this study show that review timelines of MRECs were on average more than four times as short in FTRPs. Mean number of review days of COVID-19 research from complete submission to MREC judgment was 11 days versus 52 in the RRP. In comparison, the EC of the Henan Provincial People's Hospital in China reviewed COVID-19 research even faster. Mean number of days from application of submissions until an initial review decision was 2.13 days and review of resubmission took on average 1.81 days [4]. During the EVD epidemic the WHO ERC reviewed EBV-related new submissions with an average of 15 days from complete submission to final ERC approval [6] and the MSF ERC took on average 35 (range 23-43) days from request to approval [5]. However, due to differences in composition and geography these ECs are not completely comparable.

The shortening of the review timelines found in the present study, may be explained by very motivated and flexible ad hoc (sub)committees and secretariats, the sense of urgency of those involved, full priority given to COVID-19 research, regular research put on hold due to COVID-19 measures, the use of online video meetings and administrative leniency, for example with regard to (wet) signatures. These changes did not affect the quality of the review. Moreover, the FTRP is associated with a high degree of satisfaction among both investigators as well as MREC members.

However, weaknesses faced by both investigators as MRECs were ~~a lack of~~ overview of ongoing studies and insufficient cooperation between researchers as well as MRECs of different institutions. This complicated an expedited review and a sufficient inclusion rate during a relatively short peak of the pandemic. Some institutions implemented a local COVID-19 coordinating committee to improve coordination and to protect the limited number of suitable patients from an excessive research burden. However, closer collaboration between investigators, institutions as well as MRECs is needed. This is endorsed by conclusions from previous papers [5, 6, 7, 10, 11].

The limited period of time in which sufficient patients were available for inclusion made an expedited review all the more important. Short communication lines, a designated contact person, being easily reachable, focus on essentials and increasing the sense of urgency in all involved in conducting the research were identified as factors which can contribute to an even faster review process and thus to a higher chance of reliable study outcomes. Improving these factors further could also contribute to the quality of RRP. The full priority given to COVID-19 research sometimes resulted in delayed review of (amendments to) regular research. These capacity issues should be taken into account, as was also suggested by Ma et al. [11].

In conclusion, our study provided a comprehensive overview of the fast-track review of COVID-19 research in the Netherlands during the COVID-19 pandemic. Based on the strengths and weaknesses of the FTRP described above, we have formulated recommendations for improving the review procedure in the Netherlands, as shown in table 3.

**Table 3. Recommendations**		
**Recommendation**	**Target audience**	**Review procedure**
Improve (inter)national cooperation and collaborations between investigators of different institutions	MRECs, national (research) authorities, investigators	FTRP
Implement a local coordinating committee	Institutions, MRECs	FTRP

Ensure clear communication and good telephone reachability	MRECs	FTRP and RRP
Reconsider local regulations regarding administrative review aspects and local requirements regarding submission documents	National (research) authorities	FTRP and RRP
Focus on major issues during designing, writing and reviewing research proposals	Investigators, MRECs	FTRP and RRP
Offer the possibility to attend an MREC meeting digitally	National (research) authorities, MRECs	FTRP and RRP
Investigate the possibilities of reducing workload	National (research) authorities, MRECs	FTRP

However, the way in which medical ethical review is organized and regulated by law varies internationally. Therefore, we encourage more (expedited) review procedures to be evaluated and shared internationally. This can be a first step towards more international collaboration prior to and during an epidemic, in order to make optimal use of an inclusion peak.

Acknowledgments

The authors thank the CCMO for making data available for this research. They also thank Peter van de Ven, methodologist and Caroline Terwee, methodologist, for commenting on the design of the study and Michel Paardekooper, privacy officer, for his advice on data protection. Finally they thank Richard Dekhuijzen, chair MREC CMO Arnhem-Nijmegen and chair NVMETC, and Louis Bont, chair MREC Utrecht, and Jan Swinkels, chair MREC Academic Medical Center Amsterdam, for commenting on a draft of this paper.

References

1. RIVM. Actuele informatie over het coronavirus SARS-CoV-2. Available from: https://www.rivm.nl/coronavirus-covid-19/actueel [Accessed 24th December 2020].
2. CCMO. Available from: https://english.ccmo.nl/mrecs/accredited-mrecs [Accessed 24th December 2020].
3. CCMO. Available from: https://english.ccmo.nl/investigators/primary-submission-to-the-review-committee/timelines-reviewing-committee [Accessed 24th December 2020].
4. Zhang H, Shao F, Gu J, Li L, Wang Y. Ethics Committee Reviews of Applications for Research Studies at 1 Hospital in China During the 2019 Novel Coronavirus Epidemic. *JAMA* 2020;323(18):1844-1846.
5. Schopper D, Ravinetto R, Schwartz L, et al. Research Ethics Governance in Times of Ebola. *Public Health Ethics* 2017;10(1):49-61.
6. Alirol E, Kuesel AC, Guraiib MM, de la Fuente-Núnez V, Saxena A, Gomes MF. Ethics review of studies during public health emergencies – the experience of the WHO ethics review committee during the Ebola virus disease epidemic. *BMC Medical Ethics* 2017 18(1):43.
7. Saxena A, Horby P, Amuasi J, et al. the ALERRT-WHO Workshop Ethics preparedness: facilitating ethics review during outbreaks – recommendations from an expert panel. *BMC Medical Ethics* 2019 20(1):29.
8. Creswell JW, Creswell JD. *Research Design: qualitative, quantitative, and mixed methods approaches*. 5th edition. Los Angeles: SAGE publications; 2018.
9. CCMO. CCMO-register. Available from: https://www.toetsingonline.nl [Accessed 24th December 2020].
10. Aarons D. Research in epidemic and emergency situations: A model for collaboration and expediting ethics review in two Caribbean countries. *Developing World Bioethics.* 2018 18(4):375-384.

11. Ma X, Wang Y, Gao T, et al. Challenges and strategies to research ethics in conducting COVID-19 research. *Journal of Evidence-Based Medicine*. 2020 13(2):173-177.
